# Histone chaperone HIRA complex regulates retrotransposons in embryonic stem cells

**DOI:** 10.1186/s13287-022-02814-2

**Published:** 2022-04-01

**Authors:** Miao Zhang, Xin Zhao, Xiao Feng, Xiao Hu, Xuan Zhao, Wange Lu, Xinyi Lu

**Affiliations:** 1 State Key Laboratory of Medicinal Chemical Biology, Nankai University, Tianjin, 300350 People’s Republic of China; 2 State Key Laboratory of Medicinal Chemical Biology, College of Life Sciences, Nankai University, Tianjin, 300071 People’s Republic of China

**Keywords:** Embryonic stem cell, MERVL, Ubn2, Hira, Retrotransposon

## Abstract

**Background:**

Histone cell cycle regulator (HIRA) complex is an important histone chaperone that mediates the deposition of the H3.3 histone variant onto chromatin independently from DNA synthesis. However, it is still unknown whether it participates in the expression control of retrotransposons and cell fate determination.

**Methods:**

We screened the role of HIRA complex members in repressing the expression of retrotransposons by shRNA depletion in embryonic stem cells (ESCs) followed by RT-qPCR. RNA-seq was used to study the expression profiles after depletion of individual HIRA member. RT-qPCR and western blot were used to determine overexpression of HIRA complex members. Chromatin immunoprecipitation (ChIP)-qPCR was used to find the binding of H3.3, HIRA members to chromatin. Co-immunoprecipitation was used to identify the interaction between Hira mutant and Ubn2. ChIP-qPCR was used to identify H3.3 deposition change and western blot of chromatin extract was used to validate the epigenetic change. Bioinformatics analysis was applied for the analysis of available ChIP-seq data.

**Results:**

We revealed that *Hira*, *Ubn2*, and *Ubn1* were the main repressors of 2-cell marker retrotransposon MERVL among HIRA complex members. Surprisingly, Ubn2 and Hira targeted different groups of retrotransposons and retrotransposon-derived long noncoding RNAs (lncRNAs), despite that they partially shared target genes. Furthermore, Ubn2 prevented ESCs to gain a 2-cell like state or activate trophectodermal genes upon differentiation. Mechanistically, Ubn2 and Hira suppressed retrotransposons by regulating the deposition of histone H3.3. Decreased H3.3 deposition, that was associated with the loss of *Ubn2* or *Hira,* caused the reduction of H3K9me2 and H3K9me3, which are known repressive marks of retrotransposons.

**Conclusions:**

Overall, our findings shed light on the distinct roles of HIRA complex members in controlling retrotransposons and cell fate conversion in ESCs.

**Supplementary Information:**

The online version contains supplementary material available at 10.1186/s13287-022-02814-2.

## Background

Histone chaperones are responsible for the deposition and removal of histones in chromatin. Some of the histone chaperones demonstrate histone binding selectivity towards specific histone families or variants [1]. Histone chaperones actively participate in the regulation of gene expression and chromatin epigenetics by depositing specific histone variants and safeguarding genome stability [2, 3]. They are involved in various steps of DNA replication [4, 5], DNA damage repair [6, 7], recombination [8], cell cycle checkpoint activation [9], and telomere maintenance [10–13]. They are also critical to prevent the abnormal activation of retrotransposons, including LINEs, SINE and ERVs, which can be harmful to genome stability [14]. Daxx and Atrx, as well as histone H3.3 deposited by them, silence retrotransposons in both mouse and human [14–16]. Histone chaperone of H3.1, CAF-1 complex, represses MERVL and restricts embryonic stem cells (ESCs) from the acquirement of the 2-cell like state [17–19]. The facilitates chromatin transcription (FACT) complex, which mediates the deposition of H2A/H2B histones, suppresses the MERVL and cryptic transcription in mouse ESCs [20]. Another important histone chaperone is the HIRA complex, which mediates the installment of H3.3-H4 to nucleosome [21]. It consists of Hira, Ubn1/Ubn2, and Cabin1 [22, 23]. Asf1a supplies H3.3-H4 dimers to the HIRA complex during the replication-independent histone deposition [24]. HIRA complex specifically interacts with and deposits H3.3 through subunits Ubn1 and Ubn2 whereas the Hira subunit indirectly binds to H3.3 via its interaction with Ubn1/2 [25]. In contrast to Ubn1/2 and Hira, neither Cabin1 nor Asf1a, is required for the new H3.3 deposition by HIRA complex [26–28]. HIRA deposits H3.3 and maintains proper DNA replication and rRNA production in the zygote [29]. H3.3 and its deposition histone chaperones (Daxx and Atrx) were previously found in the regulation of retrotransposons [14, 30, 31], but it is still unclear whether HIRA complex and Asf1a take part in the silencing of retrotransposons and its role in regulating ESC potency.

Here, we studied the function of members of the HIRA complex in suppressing retrotransposons. Surprisingly, different HIRA members acted distinctly in silencing retrotransposons. Ubn2 repressed LINE1 and Class III ERVs including totipotency marker MERVL [32], and Hira mainly silenced Class I and Class II ERVs. Hira was enriched on Class I and II ERVs such as IAPs, which are enriched in ESCs [33], while its depletion affected the recruitment of H3.3. In contrast, Ubn2 was enriched on Class III ERVs, while its reduction influenced H3.3 deposition and H3K9 methylation. This study extends our understandings of the function of the HIRA complex in ESCs.

## Methods

### Cell culture

Feeder-free mouse E14 embryonic stem cells (mESCs) were cultured on 0.2% gelatin (G1890, Sigma) coated plates and in standard serum/LIF medium containing 15% fetal bovine serum (FBS; SH30070.03, Hyclone) and 10 ng/ml leukaemia inhibitory factor (Z03077, GenScript). HEK 293T cells were cultured in Dulbecco's modified Eagle's medium (12100–046, Gibco) supplemented with 10% FBS (04-001-1A, Biological Industries), on 6-well plates (703001, NEST Biotechnology). Trophoblast stem cells (TSCs) induction medium contain Roswell Park Memorial Institute (RPMI) 1640 medium (01-100-1ACS, Biological Industries) supplemented with 20% FBS (SH30070.03, Hyclone), 25 ng/ml human recombinant FGF4 (Z02984, GenScript), 1 μg/ml heparin (S12004, Yuanye Biotechnology), 1 mM Sodium Pyruvate (SP0100, Solarbio), 2 mM L-glutamine (Gibco), 1% Penicillin–Streptomycin (P1400, Solarbio) and 0.1 mM *β*-mercaptoethanol (Sigma).

### RNA extraction, reverse transcription and quantitative PCR (qPCR)

To extract the total RNAs, cells were collected and lysed with 500 μl RNAiso Reagent (B9109, Takara), followed by the addition of 100 μl chloroform. After centrifugation (14,000*g* for 15 min at 4 °C), the total RNAs in the supernatant were precipitated with isopropanol and dissolved in DEPC water (B501005, Sangon Biotech). The reverse transcription was performed with 1 μg DNase-treated total RNA using the HifairII 1st Strand cDNA Synthesis Kit (11121ES60, Yeasen) based on the manufacturer's instructions. Quantitative PCR (qPCR) was performed on the qPCR detection system (CFX384 Real-Time System, Bio-Rad) using Hieff qPCR SYBR Green Master Mix (11202ES08, Yeasen). Gene expression levels were normalized to those of *Gapdh*. The relative quantitative analysis in gene expression was analyzed by the 2^−ΔΔCt^ method. All samples were examined in triplicates. Primer sequences for qPCR analysis are listed in Additional file 1: Table S1, some of which have been described previously [20, 34, 35].

### shRNA-mediated gene knockdown

For gene knockdown, short hairpin RNAs (shRNAs) for luciferase (control) or target genes were designed by an online tool (http://sirna.wi.mit.edu/) and synthesized by Tsingke Biotechnology corporation. The shRNA sequences were cloned into pSUPER-puro vector expressing a puromycin-resistant gene and purified with a kit (D6943-02, Omega). mESCs were transfected with 1 μg plasmid using Polyjet (SL100688, SignaGen), according to the manufacturer's protocol. Transfected ESCs were selected under 0.9 μg/ml puromycin from 18 to 24 h after transfection. After 3 days of selection, cells were harvested for RNA extraction. Depletion efficiency was confirmed with RT-PCR. The sequences of shRNAs are listed in Additional file 1: Table S1.

### Establishment of ESC lines

The full-length mouse *Hira* and *Ubn2* coding regions were cloned into pCAG-3HA vector (hygromycin B resistance) and pLCH72-flag (puromycin resistance). The truncation of *Hira* (*Hira* ΔWD40) containing amino acids (a.a.) 373-1016 was cloned into pCAG-3HA vector. ESCs were transfected with 1 μg respective overexpression plasmids via PolyJet reagent (SL100688, SignaGen) following the manufacturer’s recommended protocol. The ESCs were continuously selected with 800 μg/ml hygromycin B or 1 μg/ml puromycin for 2 weeks to obtain a stable cell line.

To establishment of rescue ESC lines, we synthesized the sequence of synonymous mutation of shRNAs targeting sequences of *Hira*, *Ubn1* and *Ubn2*, and constructed rescue plasmid into pCAG-3HA vector by overlap PCR. The plasmids were transfected into WT ESC and screened with 800 μg/mL hygromycin B for 2 weeks to establish stable rescue cell lines of *Hira*, *Ubn1* and *Ubn2*.

### Western blot analysis

The ESCs were primarily lysed via cell lysis buffer (0.25% Triton X-100, 10 mM EDTA, 0.1 M NaCl). The cell nuclear lysis was extracted by SDS lysis buffer and denatured. The chromatin-associated protein was prepared for the analysis of modification of H3.3. The whole-cell protein was extracted via RIPA buffer (0415A21, LEAGENE) and contained protease and phosphatase inhibitors. Proteins were separated by sodium dodecyl sulphate–polyacrylamide gel electrophoresis (SDS-PAGE) gel and then blotted onto polyvinylidene fluoride (PVDF) membrane (A29566214, GE Healthcare Life Sciences). The membrane was blocked in 5% milk and incubated with the primary antibodies overnight at 4 °C, followed by HRP-conjugated secondary antibodies for 1 h at room temperature. HRP activity was detected by Luminol HRP Substrate (WBKLS0100, Millipore). Antibodies used for western blot were as follows: anti-Flag (1:5000, F1804, Sigma), anti-HA (1:5000, 30701ES60, Yeasen), anti-H3 (1:5000, 17168-1-AP, Proteintech), anti-*β*-Tubulin (1:5000, KM9003T, Sungene Biotech), anti-*β*-Actin (1:100,000, AC026, ABclonal), anti-H3K9me2 (1:5000, ab176882, Abcam), anti-H3k9me3 (1:100,000, ab176916, Abcam), anti-Hira (1:1000, A8461, ABclonal), anti-Ubn2 (1:1000, A10516, ABclonal), anti-Asf1a (1:1000, A6528, ABclonal), anti-Ubn1 (1:1000, sc-515340, Santa Cruz), anti-Cabin1 (1:1000, sc-514269, Santa Cruz), anti-Oct4 (1:2000, sc-5279, Santa Cruz), anti-Sox2 (1:2000, sc-365964, Santa Cruz), anti-Nanog (1:2000, sc-293121, Santa Cruz). Secondary antibodies used are goat anti-rabbit IgG-HRP (sc-2004, Santa Cruz) and anti-mouse IgG-HRP (sc-516102, Santa Cruz).

### Chromatin immunoprecipitation (ChIP) coupled qPCR

For chromatin immunoprecipitation (ChIP) analysis of H3.3, Hira, Ubn2, the ESC overexpressing cells were firstly crosslinked with 1% formaldehyde and then ceased with 0.2 M glycine. Chromatin was extracted following the previous method [36]. After sonication for soluble fragments, the cell lysis was incubated with protein G magnetic beads contained Flag-tag (B26102, Bimake). The enriched chromatin DNA was extracted for qPCR analysis.

### Protein co-immunoprecipitation (co-IP)

The Flag-tag and HA-tag plasmids expressing Ubn2 or Hira were transfected into HEK-293T cells via Polyjet, and cells were lysed by cell lysis buffer supplemented with protease inhibitors. 10% of whole cell lysate was reserved for western-blot detection, and the remaining whole cell lysate was incubated with Flag-tag magnetic beads. The beads were eluted and re-suspended with the SDS-PAGE loading buffer at 98 °C for 5 min. The protein samples were used for western blot analysis.

### RNA-seq and data analysis

For sample preparation, cells were used to extract total RNA from wild-type (WT) ESC, *Hira*, *Ubn1,* and *Ubn2*-depleted ESC. For the RNA-seq library preparation, total RNA (3 μg) was used. Library samples were subjected and sequenced to obtain. Each library was pair-end sequenced for > 40 million reads by GENEWIZ. For the analysis of RNA-seq data, Trim_galore was used to trim the adapters and low-quality 3’end sequences. STAR software [37] was used for the alignment to the Grcm38 reference genome with default parameters. TE transcripts and genes were annotated according to the Ensembl database with default parameters. The expression count matrix was obtained by FeatureCounts [38]. To visualize gene expression changes, differential gene expression analysis was carried out using DESeq2. Genes with expression fold change > 1.5 and adjusted *P* value < 0.05 from DEseq2 results were used for gene ontology (GO) analysis. DAVID online analysis [39] was used for Gene Ontology (GO) term enrichment as described [40]. Gene set enrichment analysis (GSEA) was done by gseapy [41].

### Analysis of ChIP-seq data

ChIP-seq data of the enrichment of H3.3, Hira, and Ubn2 were obtained from the GEO database (GSE117034) [25] and the following analysis methods were used to process. The adapter sequences and low-quality 3′ ends of reads were removed with Trim_galore software and sequencing reads were aligned to the house mouse reference genome (mm10 assembly) using Bowtie2 [42]. ChIP-seq signal enrichment was obtained by bamCompare from Deeptools [43]. ChIP signal heatmap and profile plot were also generated by plotHeatmap and plotProfile from Deeptools [43]. ChIP-seq peak calling was done using MACS2[44]. Homer software was used to analyze the distribution of peaks.

### Statistical analysis

qPCR results were analyzed by the Student’s *t* test and statistical analyses were performed using GraphPad Prism version 9.0. *P* < 0.05 was considered statistically significant, these significant differences were defined as *P < 0.05, **P < 0.01, or ***P < 0.001.

## Results

### HIRA complex members regulate retrotransposons

**Fig. 1:**
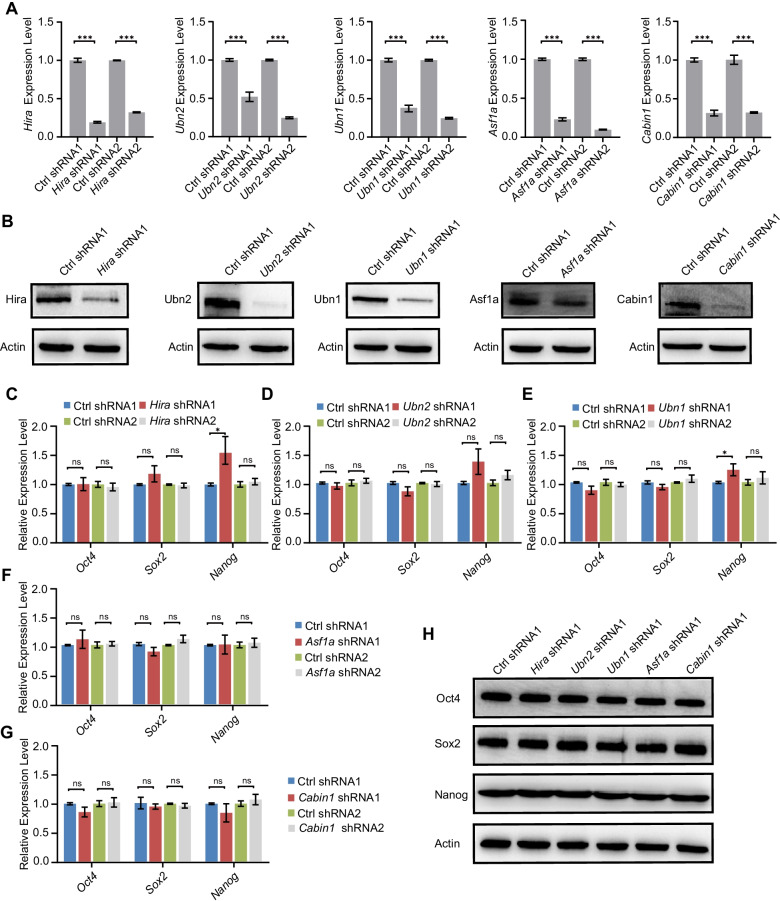
Depletion of HIRA complex members in ESCs. **a** qPCR analysis of the expression of *Hira*, *Ubn2/1*, *Asf1a,* and *Cabin1* after transfected with control shRNA and shRNAs against *Hira*, *Ubn2/1*, *Asf1a,* and *Cabin1*. The results were normalized to *Gapdh*. Data are represented as mean ± s.e.m. (*n* = 3 independent experiments). ***p < 0.001 in Student’s *t* test. **b** Western blot analysis of the indicated proteins in HIRA members and *Asf1a*-depleted ESCs. Actin was included as a loading control. **c**–**g** qPCR analysis of the expression level of pluripotency genes (*Oct4*, *Sox2,* and *Nanog*) in HIRA members *Hira* (**c**), *Ubn2* (**d**), *Ubn1* (**e**), *Asf1a* (**f**), and *Cabin1* (**g**)-depleted ESCs. The results were normalized to *Gapdh*. Data are represented as mean ± s.e.m. (*n* = 3 independent experiments). ns: non-significant, *p < 0.05 in Student’s *t* test. **h** Western blot analysis of the expression level of pluripotency genes (*Oct4*, *Sox2,* and *Nanog*) in HIRA members and *Asf1a*-depleted ESCs. Actin was included as a loading control

HIRA histone chaperone complex consists of Hira, Ubn1, Ubn2, and Cabin1, and interacts with Asf1a (Additional file 1: Fig. S1A). To study the function of members in HIRA, we depleted individual members by shRNAs. Our shRNAs could efficiently suppress the expression of HIRA complex members (Fig. 1a). The protein level of HIRA complex members could also be efficiently depleted by shRNAs (Fig. 1b). The reduction of each individual HIRA complex member did not affect the RNA expression of other members (Additional file 1: Fig. S1B–F). In addition, depletion of neither individual members of HIRA (*Hira*, *Ubn1*, *Ubn2,* and *Cabin1*) nor *Asf1a* obviously affected the expression of pluripotency genes (*Oct4*, *Sox2,* and *Nanog*) (Fig. 1c–g). Protein levels of pluripotency genes were neither influenced by the depletion of HIRA members (Fig. 1h). These results suggest that individual HIRA complex members are not required for pluripotency maintenance.

**Fig. 2:**
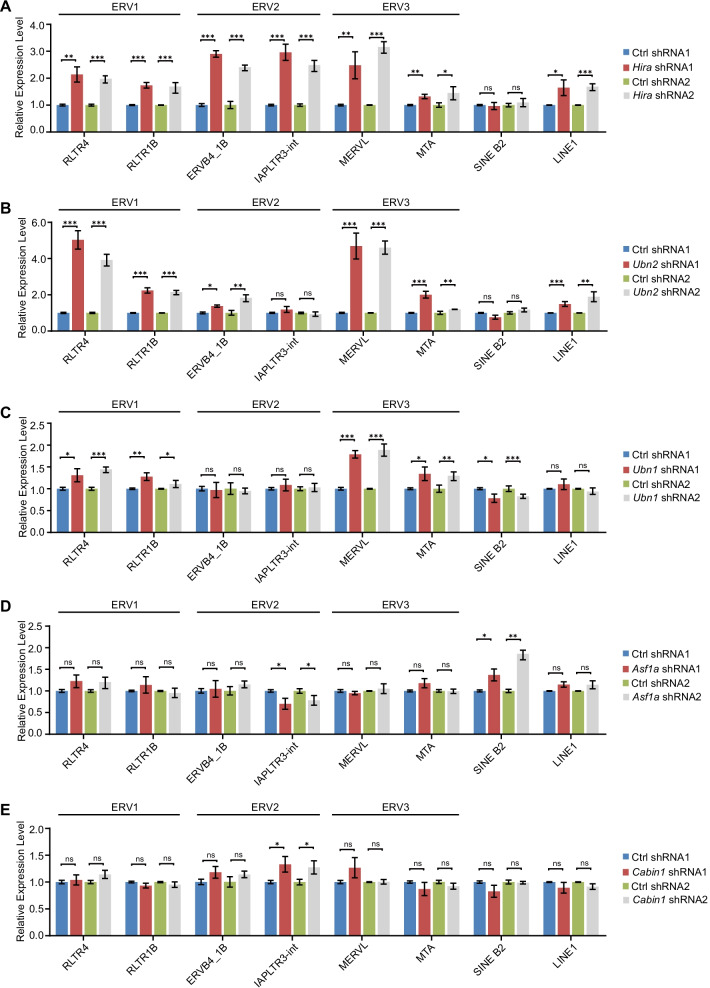
HIRA complex members regulate TE transcription. **a**–**e** qPCR analysis of different subfamilies endogenous retroviruses in *Hira* (**a**), *Ubn2* (**b**), *Ubn1* (**c**), *Asf1a* (**d**), and *Cabin1* (**e**)-depleted ESCs. The results in (**a**) to (**e**) were normalized to *Gapdh*. Data are represented as mean ± s.e.m. (*n* = 3 independent experiments) for the above qPCR results. ns: non-significant, *p < 0.05; **p < 0.01; ***p < 0.001 in Student’s *t* test

We further checked the expression of retrotransposons after depletion of HIRA complex members. The knockdown of *Hira*, *Ubn1,* or *Ubn2* resulted in the strong activation of retrotransposons to different extents (Fig. 2a–c). Interestingly, the depletion of *Ubn2* activated the highest RLTR4 and MERVL expression among different HIRA members (Fig. 2b) whereas the *Hira* decrement activated majority of retrotransposons we examined, including ERVB4_1B, IAPLTR3-int, LINE1 and MERVL (Fig. 2a). In comparison, inhibition of *Ubn1* expression led to only modest activation of retrotransposons such as RLTR4 and MERVL (Fig. 2c). The depletion of *Asf1a* or *Cabin1* affected fewer retrotransposons than *Ubn2*, *Hira* or *Ubn1* (Fig. 2d, e). *Asf1a* depletion caused slight activation of SINE B2 whereas *Cabin1* reduction weakly activated IAPLTR3-int (Fig. 2d, e). Interestingly, simultaneous depletion of *Hira* and *Ubn2* activated the both Hira- and Ubn2-targeting retrotransposons at the same time, implicating that influence of *Hira* and *Ubn2* is additive (Additional file 1: Fig. S2A, B). These data suggest that different members of the HIRA complex control distinct retrotransposon families in ESCs.

### Restoration of retrotransposon expression by overexpression of HIRA members

**Fig. 3:**
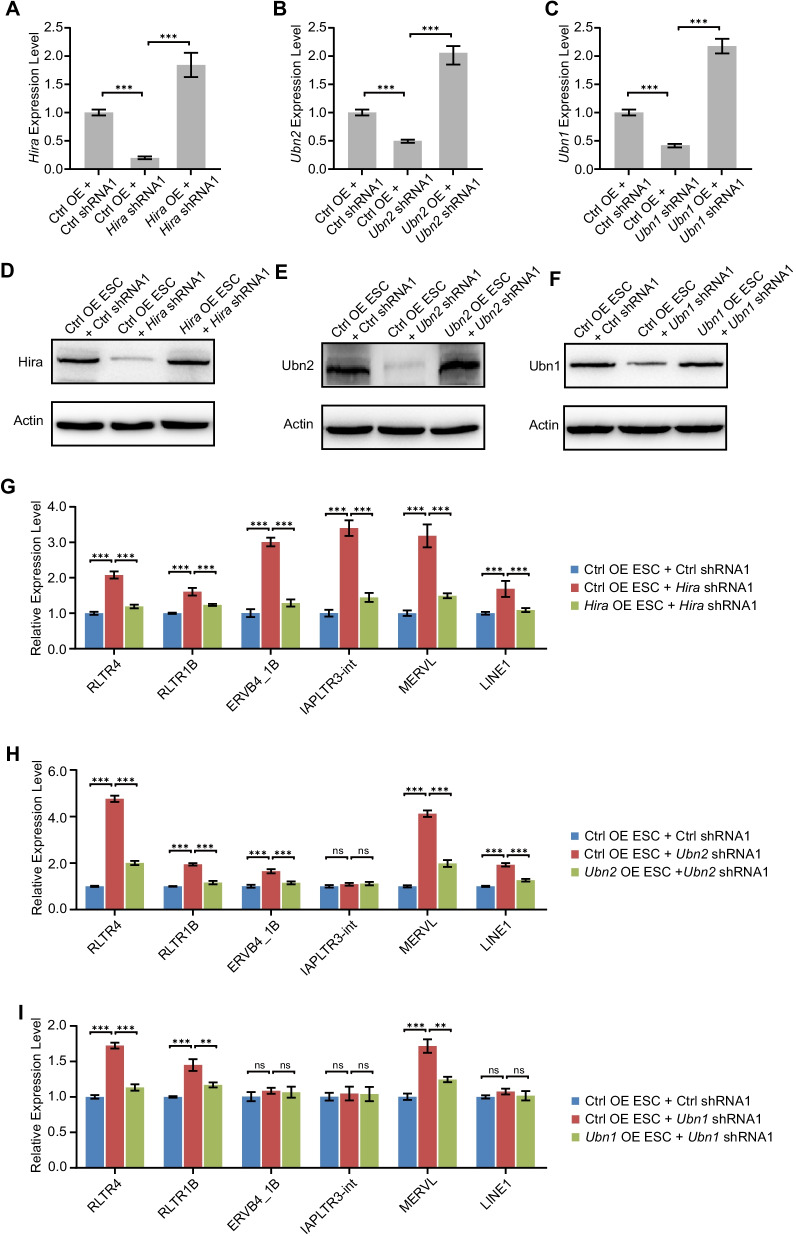
Overexpression of Hira or Ubn2 rescues the expression of retrotransposons. **a** The expression levels of *Hira* after transfected with *Hira* shRNA in control OE ESCs and *Hira* OE ESCs, as measured by RT-qPCR and normalized to *Gapdh* level; ***p < 0.001 in Student’s *t* test. **b** The expression levels of *Ubn2* after transfected with *Ubn2* shRNA in control OE ESCs and *Ubn2* OE ESCs, as measured by RT-qPCR and normalized to *Gapdh* level; ***p < 0.001 in Student’s *t* test. **c** The expression levels of *Ubn1* after transfected with *Ubn1* shRNA in control OE ESCs and *Ubn1* OE ESCs, as measured by RT-qPCR and normalized to *Gapdh* level; ***p < 0.001 in Student’s *t* test. **d**–**f** Immunoblot analysis of the expression of Hira (**d**), Ubn2 (**e**), or Ubn1 (**f**) after overexpression of Hira, Ubn2, or Ubn1. Actin was included as a loading control. **g**–**i** qPCR analysis of ERVs expression in *Hira* (**g**), *Ubn2* (**h**) and *Ubn1* (**i**) rescue ESCs lines. The results were normalized to *Gapdh*. Data are represented as mean ± s.e.m. (*n* = 3 independent experiments) for the above qPCR results. ns: non-significant, **p < 0.01; ***p < 0.001 in Student’s *t* test

To further confirm the action of *Hira*, *Ubn1* and *Ubn2*, we established ESC lines overexpressing shRNA-resistant *Hira*, *Ubn1* or *Ubn2* by generating synonymous mutations at shRNA-target loci (Additional file 1: Fig. S3A). The introduction of shRNA-resistant form of these genes could efficiently maintain the expression level of *Hira*, *Ubn1* or *Ubn2* even after the treatment of ESCs with respective shRNAs (Fig. 3a–c; Additional file 1: Fig. S3B–D). The protein level of *Hira*, *Ubn1* and *Ubn2* could also be restored by overexpression of shRNA-resistant genes (Fig. 3d–f; Additional file 1: Fig. S3B–D). The expression of retrotransposons was also rescued by expressing shRNA-resistant forms of *Hira*, *Ubn1* or *Ubn2* (Fig. 3g–i). These results futher confirm the specific action of Hira, Ubn1 and Ubn2, excluding the possibility that the activation of retrotransposons is off-target effect of shRNAs.

### Hira, Ubn2, and Ubn1 have common and shared target genes

**Fig. 4:**
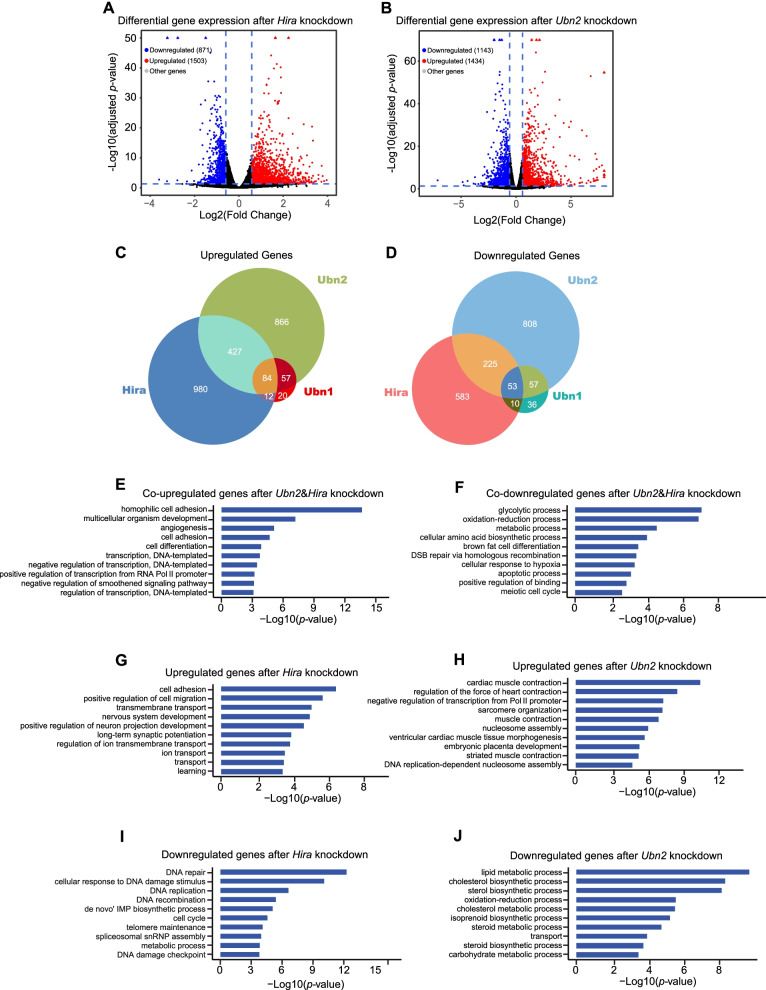
Hira and Ubn2 cooperatively regulate downstream genes in ESCs. **a**, **b** The volcano plot of gene expression in *Hira* (**a**) or *Ubn2* (**b**)-depleted ESCs versus control ESCs. Significantly upregulated genes were labeled in red and significantly downregulated genes were labeled in blue. Horizontal blue dash line marked adjusted *P* value (Wald test) 0.05 and vertical lines marked expression fold change 1.5. Triangles of (**a**) represent TEs with -log10 (adjusted *P* value) > 50. Triangles of (**b**) represent TEs with -log10 (adjusted *P* value) > 70. **c**, **d** Venn diagrams illustrated the numbers of upregulated (**c**) and downregulated (**d**) differentially expressed genes either shared or unique at all in *Hira*, *Ubn1,* and *Ubn2*-depleted ESCs. **e**, **f** Gene ontology analysis of biological processes related to co-upregulated genes (**e**) and co-downregulated genes (**f**) in *Hira* or *Ubn2*-depleted ESCs versus control ESCs. GO analysis was done with DAVID. **g**, **h** Gene ontology analysis of uniquely upregulated genes after the depletion of *Hira* (**g**) or *Ubn2* (**h**). **i**, **j** Gene ontology analysis of uniquely downregulated genes after the depletion of *Hira* (**i**) or *Ubn2* (**j**)

Given that Hira, Ubn2 and Ubn1 regulated more retrotransposons than Cabin1 and Asf1a, we subsequently performed RNA-seq to further study the function of *Hira*, *Ubn1*, and *Ubn2* in ESCs. *Hira* depletion caused the upregulation of 1503 genes and downregulation of 871 genes, whereas reduced *Ubn2* expression resulted in 1434 genes upregulated and 1143 genes downregulated (Fig. 4a, b). In contrast to the depletion of *Ubn2* and *Hira*, the disruption of *Ubn1* expression caused fewer genes with expression change (Additional file 1: Fig. S4). Since Hira, Ubn1, and Ubn2 all belong to the HIRA complex, they shared a set of common target genes (Fig. 4c, d). Among disrupted genes after depletion of *Hira* or *Ubn2*, 511 upregulated genes were common targets of both *Hira* and *Ubn2* while 278 downregulated genes were shared between *Hira* and *Ubn2* (Fig. 4c, d). Common upregulated targets of *Ubn2* and *Hira* were mainly related to cell adhesion, transcription regulation, development, and cell differentiation. Common downregulated targets of *Ubn2* and Hira were enriched of genes in metabolic pathways and oxidation–reduction (Fig. 4e, f). Different from *Ubn2* and *Hira*, most of the *Ubn1*-target genes were shared with *Ubn2* or *Hira* (Fig. 4c, d). However, *Hira* or *Ubn2* regulated the majority of genes uniquely (Fig. 4c, d). Suppression of *Hira*, but not *Ubn2*, uniquely activated gene ontology terms related to nervous system development and ion transport (Fig. 4g). This is consistent with a previous report that the loss of HIRA led to premature neural differentiation of neural progenitor cells [45]. In contrast, *Ubn2* depletion uniquely activated genes related to nucleosome assembly and muscle contraction (Fig. 4h). *Hira* depletion uniquely caused the downregulation of genes related to telomere maintenance, DNA damage, and repair (Fig. 4i) whereas unique genes downregulated by *Ubn2* depletion were enriched of terms related to various metabolic pathways (Fig. 4j). These data indicate that *Ubn2* and *Hira* have unique functions despite sharing a subset of common target genes.

### Hira and Ubn2 demonstrate specificity in regulating TEs

**Fig. 5:**
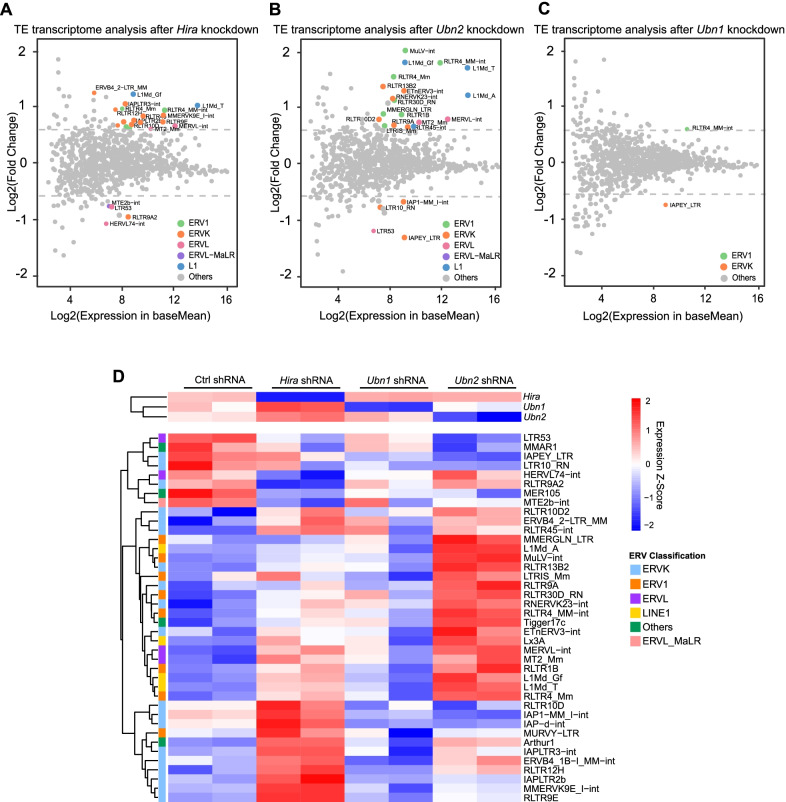
HIRA complex members regulate TE transcription genome-widely. **a**–**c** Scatter diagrams show transcriptome analysis of TE expression change after the depletion of *Hira* (**a**) or *Ubn2* (**b**) or *Ubn1* (**c**) by shRNAs. TE transcript results were used to plot these diagrams. Colored dots indicate retroelements with significant expression change (Wald test, FDR adjusted *P* value < 0.05). **d** Heatmap of RNA-Seq expression Z-scores computed for genes that are differentially expressed in *Hira*, *Ubn1,* and *Ubn2* depletion by shRNAs in ESCs versus control ESCs. Upregulated and downregulated genes are represented with red and blue colors respectively. Each column corresponds to a sample and each row corresponds to a specific gene

To further analyze the role of *Hira*, *Ubn1* and *Ubn2*, we performed RNA-seq after their depletion. RNA-seq results revealed that suppression of *Hira* and *Ubn2* released retrotransposon repression (Fig. 5a, b) and activated retrotransposon-derived lncRNA (Additional file 1: Fig. S5A, B) while the role of *Ubn1* in repressing retrotransposon was weaker than *Hira* and *Ubn2* (Fig. 5c). *Hira* mainly repressed ERVK family members whereas *Ubn2* repressed members of all three classes of ERVs and LINEs (Fig. 5d). For example, Hira specifically regulated RLTR12H and IAPLTR2b, Ubn2 regulated MERVL and MMERGLN_LTR (Fig. 5d). The above results suggest that *Hira*, *Ubn1*, and *Ubn2* have specific roles in regulating the expression of TEs and genes besides their common target genes.

### Hira and Ubn2 directly regulate retrotransposon expression and ESC fate

**Fig. 6:**
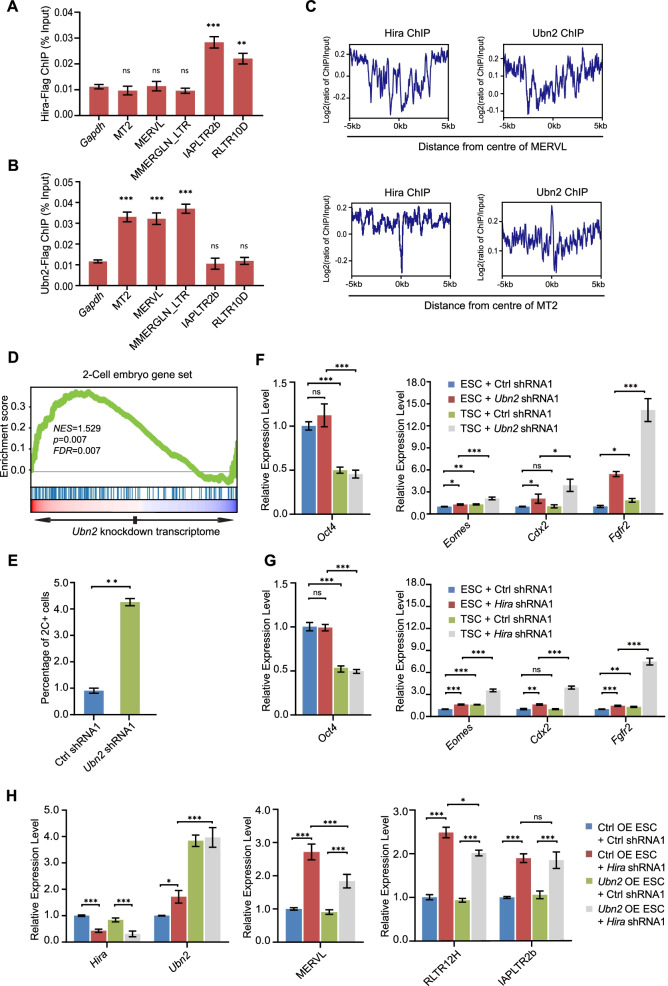
Hira and Ubn2 regulate TE expression. **a**, **b** ChIP-qPCR analysis of Hira (**a**) and Ubn2 (**b**) binding on ERV1 family member MMERGLN_LTR, ERV2 family members (IAPLTR2b and RLTR10D) and ERV3 family members (MERVL and MT2). Biological triplicate data (*n* = 3 extracts) are presented as mean ± s.e.m. ns: non-significant, **p < 0.01; ***p < 0.001 in Student’s *t* test. **c** Hira and Ubn2 binding profile around the center of MT2 locus (MERVL-LTR) respectively. The ChIP-seq signal was calculated as the log2 ratio of the normalized number of reads relative to the input. **d** GSEA analysis of upregulated genes after *Ubn2* knockdown for the enrichment of 2C genes. Red, upregulated genes; blue, downregulated genes; NES, normalized enrichment scores; FDR, false discovery rate. The Kolmogorov–Smirnov statistic was used for the calculation of the *P* value. **e** Flow cytometry analysis of the 2C^+^ population in 2C::tdTomato reporter ESCs after depletion of *Ubn2* by shRNA. Data are represented as mean ± s.e.m. (*n* = 3 independent experiments). **p < 0.01 in Student’s *t* test. **f**, **g** The expression level of pluripotent gene *Oct4* and trophectoderm markers (*Eomes, Cdx2,* and *Fgfr2*) in *Ubn2* (**f**) and *Hira* (**g**)-depleted ESCs cultured under ESC or TSC medium for 3 days, as measured by RT-qPCR and normalized to *Gapdh* levels. Data are represented as mean ± s.e.m. (*n* = 3 independent experiments). ns, non-significant; *p < 0.05; **p < 0.01; ***p < 0.001 in Student’s *t* test. **h** qPCR analysis of *Hira*, *Ubn2*, MERVL, and ERV2 family member (RLTR12H and IAPLTR2b) after *Hira* depletion in *Ubn2* overexpression ESCs. The results were normalized to *Gapdh*. Data are represented as mean ± s.e.m. (*n* = 3 independent experiments). ns: non-significant, *p < 0.05; ***p < 0.001 in Student’s *t* test

Since only Hira and Ubn2 mainly participate in the repression of ERVs, we further studied whether Hira and Ubn2 directly regulated the expression of ERVs. We first performed ChIP-qPCR to test the enrichment of Hira and Ubn2 on ERVs. We found that Hira was enriched on IAPLTR2b and RLTR10D, but not on MT2/MERVL or MMERGLN_LTR (Fig. 6a). In contrast, Ubn2 was more enriched on MT2/MERVL and MMERGLN_LTR (Fig. 6b). Analysis of published ChIP-seq data confirmed that only Ubn2 but not Hira was enriched on MT2/MERVL (Fig. 6c; Additional file 1: Fig. S6A), suggesting Ubn2 as a direct repressor of MERVL. Since MERVL is a marker of 2-cell like cells, we also found enrichment of 2-cell genes in *Ubn2*-depleted ESCs (Fig. 6d) as well as increment of percentage of MERVL-gag + 2C-like cells within *Ubn2*-depleted population (Fig. 6e). We examined the differentiation capacity of ESCs after *Ubn2* depletion. We found that *Ubn2* depletion could induce ESCs to further upregulate trophectodermal marker genes (*Eomes*, *Cdx2,* and *Fgfr2*) upon directed differentiation to TSCs (Fig. 6f). Likewise, *Hira* depletion upregulated trophectodermal marker genes but to a lesser extent (Fig. 6g). To examine how Hira also regulates MERVL, we established cell lines overexpressing *Ubn2*. After overexpressing *Ubn2*, *Hira* depletion was partially rescued (Fig. 6h). Furthermore, overexpressing a mutant *Hira*, which cannot interact with Ubn2 (Additional file 1: Fig. S6B, C), rescued the expression of ERVB4_1B and RLTR12H, but not MERVL (Additional file 1: Fig. S6D). These results support that Ubn2 directly suppresses MERVL and 2-cell fate determination while Hira may regulate MERVL through its interaction with Ubn2.

### Hira and Ubn2 act through deposition of H3.3 and H3K9 methylation

**Fig. 7:**
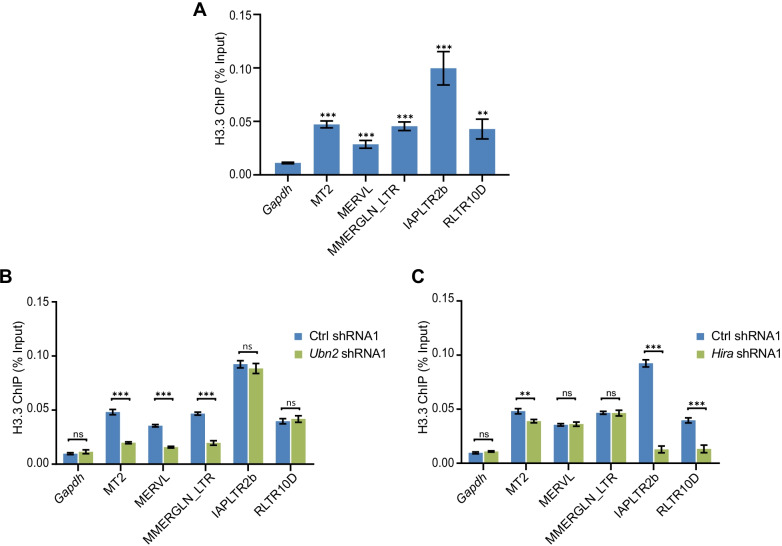
Hira and Ubn2 regulate H3.3 deposition. **a** ChIP-qPCR analysis of H3.3 binding on three families of ERVs (ERV1: MMERGLN_LTR; ERV2: IAPLTR2b and RLTR10D; ERV3: MERVL and MT2). **p < 0.01; ***p < 0.001 in Student’s *t* test. **b**, **c** ChIP-qPCR analysis showed that the binding effects of H3.3 on ERV1 and ERV3 were significantly attenuated in *Ubn2*-depleted ESCs (**b**), and the binding effect on ERV2 family members (IAPLTR2b and RLTR10D) was weakened after *Hira* depletion (**c**). Data are represented as mean ± s.e.m. (*n* = 3 independent experiments) for the above ChIP-qPCR results. ns: non-significant, **p < 0.01; ***p < 0.001 in Student’s *t* test

Next, we investigated the functional mechanism of Hira and Ubn2 in repressing retrotransposons. Given that histone H3.3 is deposited by the HIRA complex, we examined the H3.3 enrichment on ERVs with an ESC line overexpressing HA-tagged H3.3. Indeed, histone H3.3 was enriched on all kinds of ERVs bound by Hira and Ubn2 (Fig. 7a). Hence, we next tested the deposition of H3.3 after *Hira* or *Ubn2* depletion. The depositions of H3.3 on MT2/MERVL and MMERGLN_LTR were prominently affected by *Ubn2* depletion, but no effect was observed on IAPLTR2b and RLTR10D (Fig. 7b). In addition, H3.3 deposition was greatly affected on IAPLTR2b and RLTR10D after *Hira* depletion (Fig. 7c). However, the impact of *Hira* depletion on MT2/MERVL is much weaker (Fig. 7c) whereas there was no effect on MMERGLN_LTR. This is expected because *Hira* suppression activated MT2/MERVL but not MMERGLN_LTR (Fig. 5d). Together, these findings suggest that Ubn2 and Hira suppress retrotransposons by controlling the installment of H3.3.

**Fig. 8:**
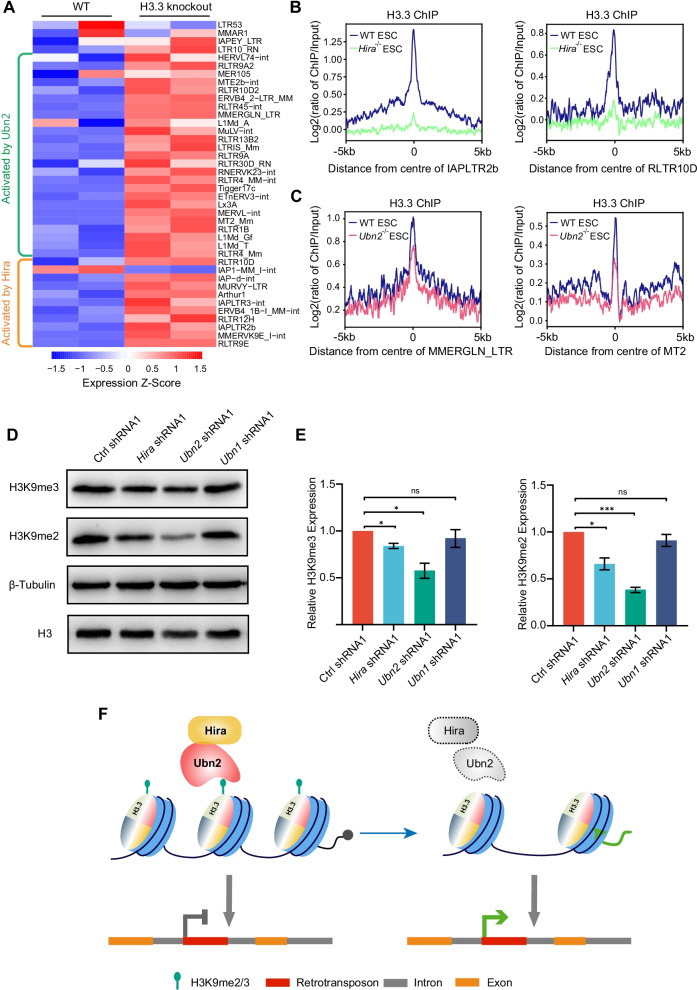
The reduction of H3K9me2/3 after *Hira* or *Ubn2* depletion. **a** Heatmap of RNA-Seq expression Z-scores computed for genes that are differentially expressed between WT ESCs and *H3.3* knockout ESCs. The left side shows the genes regulated by Hira and Ubn2 respectively. The upregulated and downregulated genes are represented with red and blue colors, respectively. Each column corresponds to a sample and each row corresponds to a specific gene. **b** ChIP-seq enrichment of H3.3 around the center of IAPLTR2b or RLTR10D locus in WT ESCs (blue) and *Hira*^−/−^ ESCs (green). The ChIP-seq signal was calculated as the log2 ratio of the normalized number of reads relative to the input. The published ChIP-seq data is from GEO: GSE117034. **c** ChIP-seq enrichment of H3.3 around the center of MMERGLN_LTR or MT2 locus in WT ESCs (blue) and *Ubn2*^−/−^ ESCs (red). The ChIP-seq signal was calculated as the log2 ratio of the normalized number of reads relative to the input. The data of ChIP-seq are available at GEO: GSE117034. **d** Western blot analysis of H3, *β*-Tubulin, H3K9me2 and H3K9me3 protein levels in ESCs transfected with shRNAs against target genes (*Hira*, *Ubn2*, and *Ubn1*) or control shRNA respectively. *β*-Tubulin was included as a loading control. **e** The relative protein level of H3K9me2 and H3K9me3 was obtained according to western blot band in (**d**). Gray scanning analysis was normalized to that of *β*-Tubulin. Data are represented as mean ± s.e.m. (*n* = 3 independent experiments). ns: non-significant, *p < 0.05; ***p < 0.001 in Student’s *t* test. **f** Schematic of Hira and Ubn2 function in the repression of retrotransposons. In WT ESCs, Hira and Ubn2 mediate the placement of H3.3 and H3K9 methylation, and thus repress the expression of retrotransposons. In the absence of *Ubn2* or *Hira*, retrotransposon-associated H3.3 and H3K9me2/3 reduce, and thus activate the expression of retrotransposons

Furthermore, we analyzed published RNA-seq and ChIP-seq data to confirm our analysis. H3.1/3.2 was only strongly enriched on ERV3 member MT2/MERVL, however, H3.3 was enriched on all three classes of ERVs (Additional file 1: Fig. S7A–D). H3.3-deficient ESCs demonstrated upregulation of almost all TEs targeted by Ubn2 and Hira (Fig. 8a). Moreover, by analysis of published H3.3 ChIP-seq data after the knockout of *Ubn2* or *Hira* [25], H3.3 was removed from target TEs (Fig. 8b, c). *Ubn2* depletion led to the reduction of H3K9me2 and H3K9me3 (Fig. 8d, e). However, *Hira* depletion resulted in a milder effect on H3K9me2 and H3K9me3 (Fig. 8d, e). We further analyzed published ChIP-seq data and found increased enrichment of histone marks related to expression activation (H3K4me1, H3K27ac, H3K64ac, and H3K122ac) on MERVL (Additional file 1: Fig. S8A–D). These results suggest that H3.3 marked by H3K9me2/3 are critical to ERV silencing after *Hira* or *Ubn2* depletion.

## Discussion

In summary, we proposed a model for which Hira and Ubn2 in regulating retrotransposon expression (Fig. 8f). In the presence of the HIRA complex, HIRA interacts with H3.3 and mediates the placement of H3.3 and the associated H3K9 methylation on retrotransposons to repress their expression whereas in the absence of Ubn2 or Hira, H3.3 and H3K9me2/3 on retrotransposons are removed to allow the expression of retrotransposons (Fig. 8f).

HIRA complex is often considered to function together in regulating gene expression [46]. Hira is the scaffold protein of the complex and it binds to H3.3 via Ubn1/2 [25]. Asf1 protein is known to supply H3.3-H4 dimer to HIRA complex [24] where as Cabin1 is involved in the formation of heterochromatin in senescent cells [47]. It is intriguing to see different members of the HIRA complex regulate distinct ERV families. *Asf1a* and *Cabin1* are not involved in suppressing retrotransposon expression (Fig. 2d, e). It appears that *Ubn2* and *Hira* played more important roles than *Ubn1* in repressing retrotransposon expression (Fig. 2a–c). Previous studies suggest the specific role of these members within HIRA complex besides function as a whole. Cabin1 has its own specific targets in addition to shared target genes with Hira [47]. Besides working with HIRA complex, Asf1 also supplies H3-H4 dimers to CAF1 complex [48]. Ubn1 and Ubn2 are paralogous genes but alternatively present in the HIRA complex [25], forming two independent complexes Hira-Ubn1 and Hira-Ubn2. Previously, it was found that simultaneous depletion of *Ubn1* and *Ubn2* caused more severe loss of H3.3 than *Hira* knockout [25], suggesting that Ubn1/2 have Hira-independent functions in depositing H3.3 in ESCs. Hence, it is possible that Ubn1 and Ubn2 contribute to the selection of target loci for the HIRA complex. One possible route for Ubn1/2 and Hira to target specific loci is through interacting with different protein partners. The interacting protein of HIRA can modulate the recruitment or the function of HIRA. For example, replication protein A (RPA) complex interacts and recruits HIRA to deposit newly synthesized H3.3 at RPA binding region [49]. Interaction of Asf1b with HIRA facilitates transcription restart after DNA damage repair [50]. Furthermore, previous studies have found HIRA worked with other protein partners to repress gene expression [51, 52]. For example, Ubn2, Hira and Cabin1, not Ubn1, interact with Zbtb2 in ESCs [53]. In the absence of Ubn1/2, Hira still can interact with other protein partners such as SRCAP [54]. These findings prove that it is possible for HIRA members to gain specificity through interacting with partner proteins. It will be interesting to investigate which factors render Ubn1/2 and Hira the specificity to recognize different classes of retrotransposons.

Another possible route to achieve target specificity is that different HIRA complex members take part in distinct processes. HIRA complex can regulate H3.3 deposition in two independent pathways. Hira trimerization and Ubn1 are required for the deposition of newly synthesized H3.3 while Asf1 is not required [55]. In contrast, recycling of old H3.3 requires Hira and Asf1 but not Ubn1/2 or Hira trimerization [55]. Hence, we speculate that Hira may repress its target retrotransposons in a complex without the need of Ubn1/2 in a similarly manner, possibly through regulating the recycling of old H3.3. This is supported by the fact that overexpressing Hira mutant without WD40 domain, which is responsible for interacting with Ubn1/2 [23], can still rescue the expression of Hira-target retrotransposons after the depletion of *Hira* with shRNA against WD40 domain (Additional file 1: Fig. S6D), suggesting that Ubn1/2 is not required to repress Hira-target transposons. The binding of Hira not Ubn2 on IAPLTR2b and RLTR10D supports that Hira represses these retrotransposons in a complex without Ubn2 (Fig. 6a, b). However, Ubn2 may function together with Hira in the same complex, given that a portion of target retrotransposons of Ubn2 were partially derepressed by *Hira* depletion (Fig. 5d). The specific enrichment of Ubn2 on target retrotransposons can be explained by the fact that Ubn1/2 directly interacts with H3.3 and DNA in contrast to the indirect Hira-H3.3 interaction, which may not stable enough to be detected by ChIP. It is possible that *Hira* depletion disrupts *Ubn2* targets by disrupting the H3.3 deposition activity of HIRA complex or influencing Ubn2 stability or activity, given that the scaffold role of Hira in the complex is able to organize the partner proteins and stabilize Ubn2 protein [50]. Given the role of Hira-Ubn1/2 in depositing new H3.3 [55], it is likely that Ubn2-Hira controls retrotransposons through modulating the localization of newly synthesized H3.3.

Besides the HIRA complex, histone chaperone CAF-1 and FACT were also shown to repress MERVL and 2-cell fate in ESCs [17–20]. H3.3 and Hira were found to repress 2-cell fate as well [56]. However, it is unclear why CAF-1 and FACT mainly regulate MERVL but HIRA complex represses MERVL and other ERVs at the same time. One possibility is that FACT controls MERVL through H2A/H2B but HIRA works through H3.3 [20]. However, the extent of contribution to ERV repression from each histone member is still unclear. CAF-1 mediates DNA synthesis-coupled H3.1 incorporation, but HIRA can mediate H3.3 deposition at the DNA replication site if H3.1 deposition is impaired [26]. Consistently, we observed the enrichment of both H3.3 and H3.1/H3.2 on MERVL (Additional file 1: Fig. S7D). However, for other ERVs regulated by HIRA, there was only enrichment of H3.3 (Additional file 1: Fig. S7A–C). It could be due to H3.3 is present throughout the cell cycle while H3.1 deposition is only coupled to DNA replication [57]. It will be intriguing to check whether the activated MERVL after the loss of CAF-1 or HIRA complex is from different cell cycle phases.

It is interesting to see that MERVL was activated after *Ubn2* depletion (Figs. 2b, 5d). MERVL marks 2-cell like cell population, suggesting a role of Ubn2 in pluripotency to totipotency conversion. In consistency with above finding, IAPEy, which is a marker of naïve ESCs [33], was downregulated after *Ubn2* depletion (Fig. 5d). These results imply that *Ubn2* depletion repressed naïve ESC state but activated totipotent 2-cell like state.

## Conclusions

In conclusion, we found that different members of the HIRA complex demonstrated preferences in repressing retrotransposons in ESCs. Our study shed light on the loci-specific activity of HIRA and H3.3 in gene expression regulation and a role of the HIRA complex in regulating ESC state determination.

## Supplementary Information

**Additional file 1**. The supplementary figures S1-8 and corresponding figure legends.

## Data Availability

Sequencing data generated from our study have been deposited in Gene Expression Omnibus under GSE177026 for RNA-seq (https://www.ncbi.nlm.nih.gov/geo/query/acc.cgi?acc=GSE177026). RNA-seq data of *H3.3*^−/−^ ESC were obtained from the GEO database (GSE114549) [58] and processed with the above flow. ChIP-seq data of the enrichment of H3.3 and H3.1/2 were obtained from the GEO database (GSE59188) [14] and H3.3 Related epigenetic modifications were obtained from the GEO database (GSE114548) [58]. ChIP-seq data of H3.3, Hira, and Ubn2 were from GEO database (GSE117034) [25].
