# Supplementary material for: Histone chaperone HIRA complex regulates retrotransposons in embryonic stem cells
Source: Stem Cell Res Ther. 2022 Apr 1;13:137. doi: 10.1186/s13287-022-02814-2 (PMC8973876; doi:10.1186/s13287-022-02814-2)
Supplement: Supplementary file 1 — Additional file 1. The supplementary figures S1-8 and corresponding figure legends. [file 13287_2022_2814_MOESM1_ESM.pdf]

Supplementary Information

Supplementary Figure S1-8

Supplementary Table S1

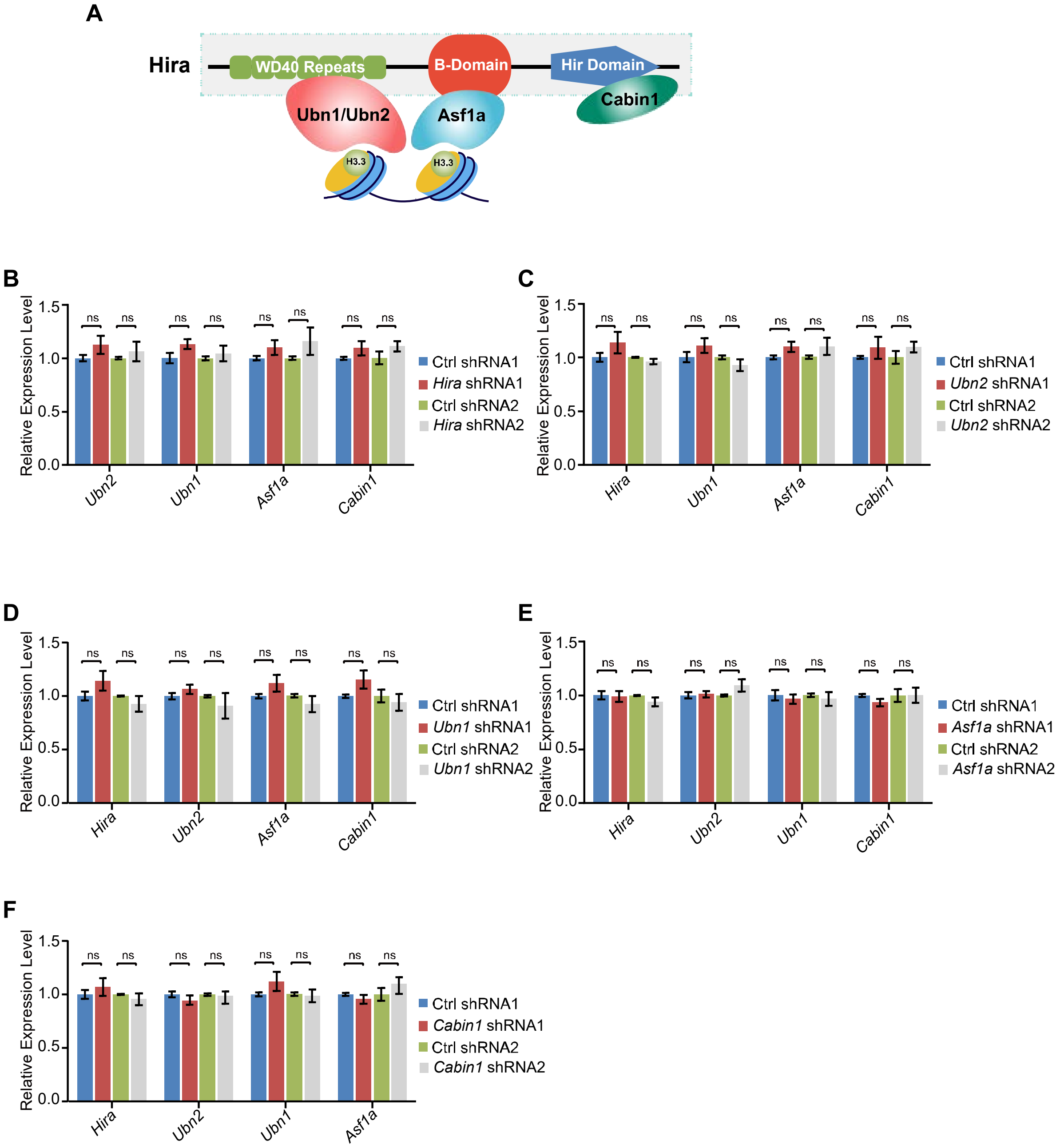

Fig. S1. The depletion of components of Hira complex does not disturb each other at mRNA level

(A) Schematic diagram of the interrelation of HIRA members. Hira subunit associates with H3.3 through Ubn1/2 and Asf1a via its WD40 domain and B domain respectively.

(B) qPCR analysis of the expression of *Ubn2*, *Ubn1, Asf1a,* and *Cabin1* after transfected with control shRNA and shRNA against *Hira*.

(C) qPCR analysis of the expression of *Hira*, *Ubn1, Asf1a,* and *Cabin1* after transfected with control shRNA and shRNA against *Ubn2*.

(D) qPCR analysis of the expression of *Hira*, *Ubn2, Asf1a,* and *Cabin1* after transfected with control shRNA and shRNA against *Ubn1*.

(E) qPCR analysis of the expression of *Hira*, *Ubn2, Ubn1,* and *Cabin1* after transfected with control shRNA and shRNA against *Asf1a*.

(F) qPCR analysis of the expression of *Hira*, *Ubn2, Ubn1,* and *Asf1a* after transfected with control shRNA and shRNA against *Cabin1*. The results in B to F were normalized to *Gapdh*. Data are represented as mean ± s.e.m. (n = 3 independent experiments) for the above qPCR results. ns: non-significant in Student's *t*-test.

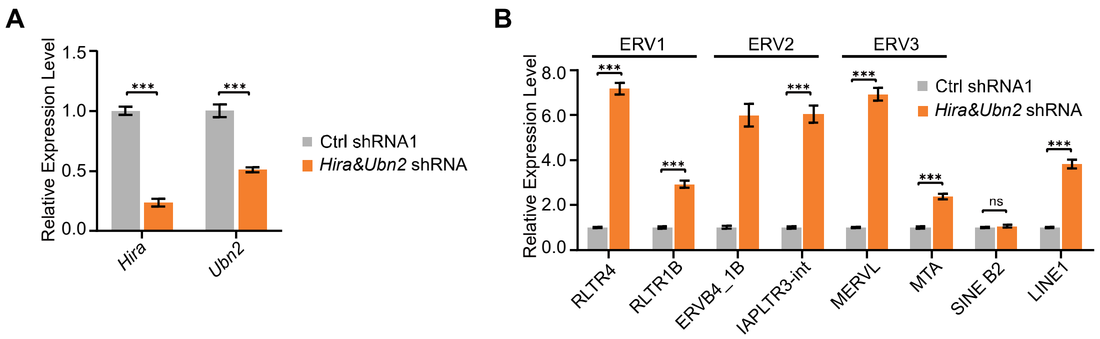

Fig. S2. *Hira* and *Ubn2* double-knockdown in ESCs.
(**A**) qPCR analysis of the expression of *Hira* and *Ubn2* of double-knockdown both *Hira* and *Ubn2* by shRNA in ESCs at the same time. ***$p < 0.001$ in Student's t-test.
(**B**) qPCR analysis of the expression of different subfamilies endogenous retroviruses in *Hira* & *Ubn2* shRNA in ESCs. ns: non-significant, ***$p < 0.001$ in Student's t-test.

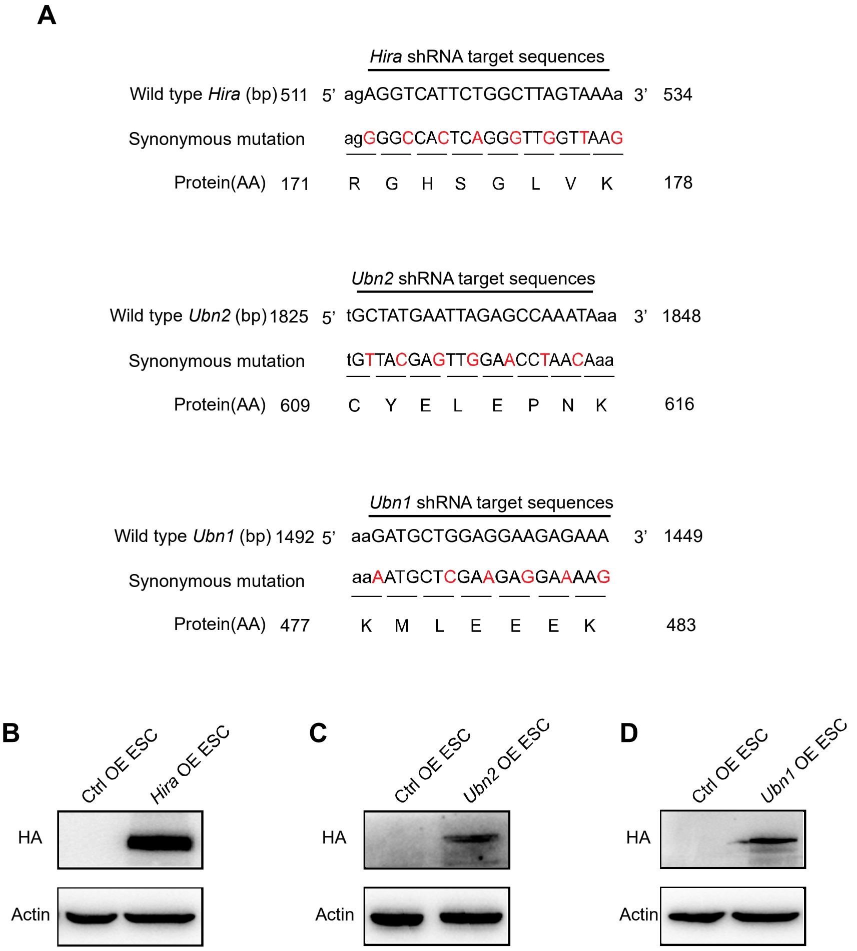

Fig. S3. Overexpression of shRNA-resistant *Hira*, *Ubn2*, and *Ubn1* in ESCs.

(A) A schematic of shRNAs targeting sequences, synonymous mutation sequences and corresponding protein of *Hira*, *Ubn2* and *Ubn1*. Mutated nucleotide is highlighted in red; positions of nucleotide in gene are indicated on top.

(B-D) Western blot analysis of Hira (B), Ubn2 (C), and Ubn1 (D) from overexpression ESCs with anti-HA antibody. Actin was included as a loading control.

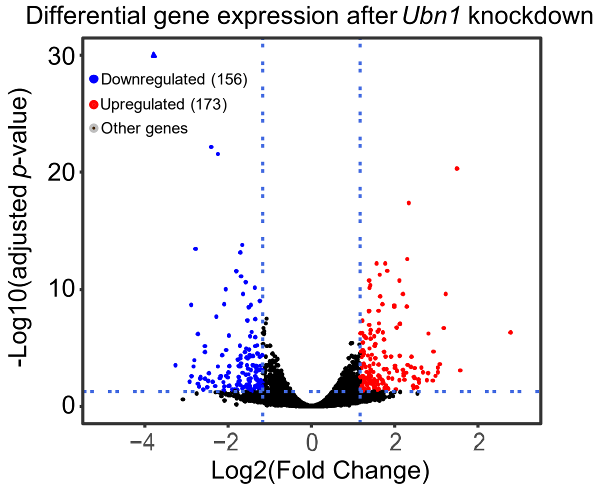

Fig. S4. Genome-wide expression changes after the knockdown of *Ubn1*.

The volcano plot of gene expression in *Ubn1*-depleted ESCs versus control ESCs. Significantly upregulated genes were labeled in red and significantly downregulated genes were labeled in blue. Horizontal blue dash line marked adjusted *P*-value (Wald test) 0.05 and vertical lines marked expression fold change 1.5. Triangles represent TEs with -log10 (adjusted *P*-value) > 30.

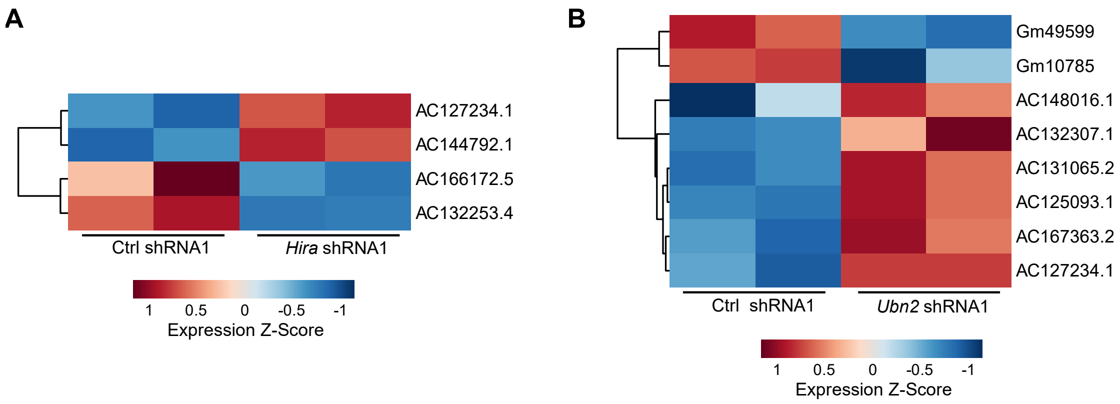

Fig. S5. Hira and Ubn2 regulate the expression of transposon-derived lncRNAs. (A-B) Heatmap of RNA-Seq expression Z-scores for transposon-derived lncRNAs that are differentially expressed in *Hira* (A) and *Ubn2* (B)-depleted ESCs versus control ESCs. Upregulated and downregulated genes are represented with red and blue colors respectively.

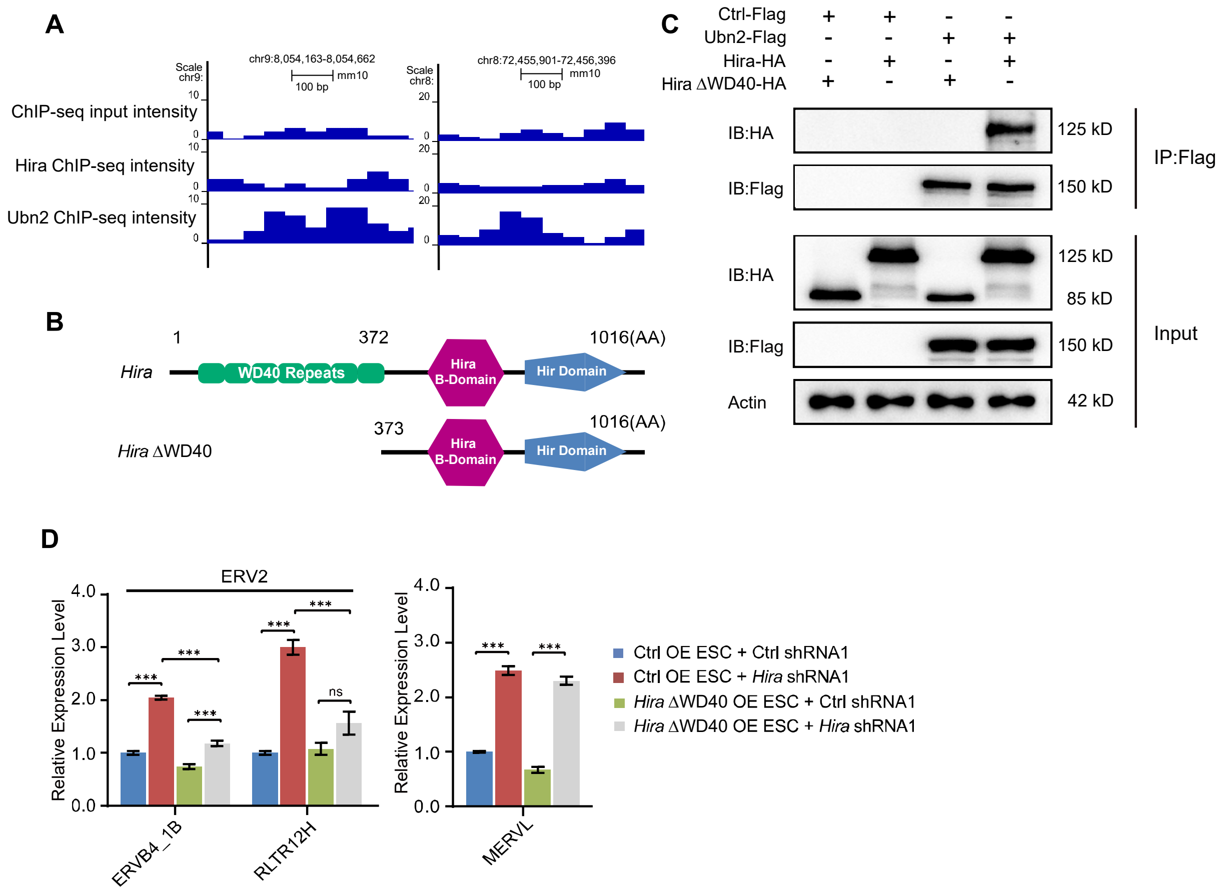

Fig. S6. Hira regulates MERVL through the interaction with Ubn2.

(A) According to the published ChIP-seq data described in Methods, the enrichment of Hira and Ubn2 in the MT2/MERVL region is exemplified. Inputs are included as controls.

(B) A schematic summary of *Hira* ΔWD40 mutant used for rescue. The length of the WD40 mutant form is indicated at the top in amino acids (AA). Δ, deletion.

(C) Co-immunoprecipitation results confirmed that Ubn2 combined with Hira at its WD40 domain. The plasmids of Ctrl-Flag/*Ubn2*-Flag/*Hira*-HA/*Hira* ΔWD40-HA were respectively transfected into HEK 293T cells and analyzed via western blot of anti-Flag immunoprecipitation.

(D) qPCR analysis of ERVB4_1B, RLTR12H, and MERVL in *Hira*-depleted ESCs after overexpression of *Hira* ΔWD40. qPCR results are normalized to *Gapdh*. Data are represented as mean ± s.e.m. (n = 3 independent experiments). ns: non-significant, ***$p < 0.001$ in Student's t-test.

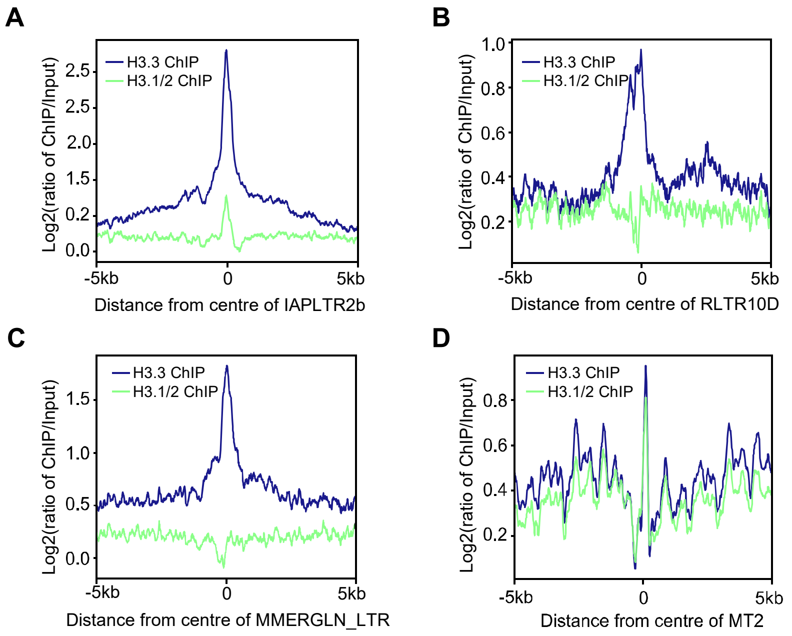

Fig. S7. H3.3 is enriched on three classes of ERVs.

(A-D) H3.3 (blue) and H3.1/H3.2 (green) binding profile around the center of IAPLTR2b (A), RLTR10D (B), MMERGLN_LTR (C), and MT2 (D) locus in ESCs. The ChIP-seq signal was calculated as the log2 ratio of the normalized number of reads relative to the input.

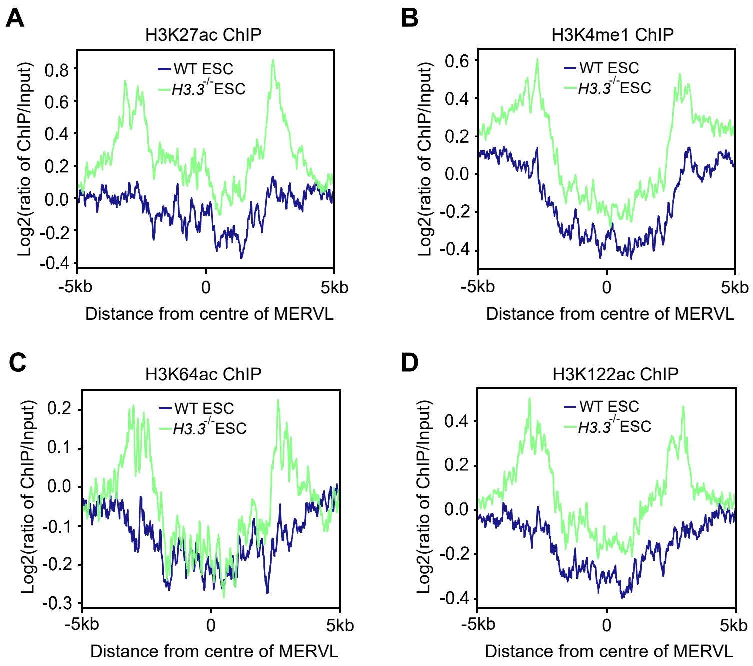

Fig. S8. Enrichment of histone marks on MERVL after *H3.3* knockout.
(A-D) Several histone marks (H3K27ac (A), H3K4me1 (B), H3K64ac (C), H3K122ac (D)) binding profile around the center of MERVL locus in WT ESCs (blue) and *H3.3*$^{-/-}$ ESCs (green). The ChIP-seq signal was calculated as the log2 ratio of the normalized number of reads relative to the input.

Table S1. Sequences of primers and shRNAs

Gene	Sequence F	Sequence R	Reference
Gapdh	AGAAACCTGCCAAGTATGATGAC	GTCATTGAGAGCAATGCCAG	Chen, et al. (2020) Nucleic acids res, 48, 10211-10225.
Oct4	GTGGAAAGCAACTCAGAGG	GGTTCCACCTTCTCCAACT	Chen, et al. (2020) Nucleic acids res, 48, 10211-10225.
Sox2	GCGGAGTGGAAACTTTTGTCC	CGGGAAGCGTGTACTTATCCTT	Chen, et al. (2020) Nucleic acids res, 48, 10211-10225.
Nanog	TTGCTTACAAGGGTCTGCTACT	ACTGGTAGAAGAATCAGGGCT	Chen, et al. (2020) Nucleic acids res, 48, 10211-10225.
Hira	TGGTCGGAGGAGAATCACG	GAGGGTGACGATGCAGCAG	
Ubn1	CTATGCCTGAGCAGGTAGCC	GATCTTCACCACCTGGCACA	
Ubn2	CTGCCTCAGGGTCTTCAGTG	CCCAGCATCCCAAAAGGAGT	
Asf1a	CACCGAATGCAGGACTCATC	GCATCTGTTGAAAGAAGGGACTG	
Cabin1	TCGCCACTCAGACTTGGAAC	TAGTGGGAGCAGCAGTTGTG	
Cdx2	AGGCTGAGCCATGAGGAGTA	TGAGGTCCATAATTCCACTCA	
Eomes	CAATGTTTTCGTGGAAGTGG	GTTAGGAGATTCTGGGTGAA	
Fgfr2	CCTCGATGTCGTTGAACGGTC	CAGCATCCATCTCCGTCACA	
MERVL	AAGAGCCAAGACCTGCTGAG	TCCTCGTTTCTGCAACTGGT	Zhang, et al. (2019) Nucleic acids res, 47, 8485-8501.
MT2	CTCTACCACTTGGACCATATGAC	GAGGCTCCAAACAGCATCTCTA	Zhang, et al. (2019) Nucleic acids res, 47, 8485-8501.
SINEB1	GTGGCGCACGCCTTTAATC	GACAGGGTTTCTCTGTGTAG	Chen, et al. (2020) Nucleic acids res, 48, 10211-10225.
SINEB2	GAGTAAGAGCACCCGACTGC	AGAAGAGGGAGTCAGATCTCGT	Chen, et al. (2021) Stem cells int, 2021, 6657597.
LINE1	GGACCAGAAAAGAAATTCCTCCCG	CTCTTCTGGCTTTCATAGTCTCTGG	Chen, et al. (2021) Stem cells int, 2021, 6657597.
RLTR1B	GGTCCACACAAACACCTACCTT	TTTGAGATACACCCTTCGAGGT	Zhang, et al. (2019) Nucleic acids res, 47, 8485-8501.
MTA	TCTGTGGGATGTTGTGTAGGAG	CCACAGATCTTCACAATCCAAA	Zhang, et al. (2019) Nucleic acids res, 47, 8485-8501.
IAPLTR2b	CACATTCGCCGTTACAAGAT	TTGCTTACATCTTCAGGAGC	
MMERGLN_LTR	GAGCTTTGAAACCTGGGGCT	AAACATCAGCAGCCTGTAAC	
RLTR12H	GCTGAACAGCCAATGACTGG	CATGCCCGACCTCATGGCGA	
RLTR10D	GACTGCAGCCAAGTCTTATG	TCAGCCCAGTCCGCGTAACA	
RLTR4	AGCGTTAATTTGGTCAAAGTCT	CCAAGTATTGGGGACTGATAAT	
ERVB4 1B	ATGGAGATATTCTTAGCTCTG	GAATTGACAGACATATGGAC	
IAPLTR3-int	GCGGTACAAGACTGGCTTAA	GAACAGCTCCTCTTGCACGT	
MT2 ChIP-qPCR	GGCTACACCTTCTGCTGGAG	TCGCAGCTGTGAATGGAAGT	

Gene	shRNA Sequence
Control shRNA	GATGAAATGGGTAAGTACA
Hira shRNA1	AGGTCATTCTGGCTTAGTAAA
Hira shRNA2	CAGGACCGTTAGCCATAAT
Ubn1 shRNA1	GATGCTGGAGGAAGAGAAA
Ubn1 shRNA2	CGGAAGAAATTCCAGTGGAAT
Ubn2 shRNA1	GCTATGAATTAGAGCCAAATA
Ubn2 shRNA2	GGTGCTACTAAACCGTTGT
Asf1a shRNA1	GTGGGCTCTGCAGAAAGTGAA
Asf1a shRNA2	GGGTAACAGTTGTTCTGAT
Cabin1 shRNA1	GACCACGATTACGTCAAAT
Cabin1 shRNA2	GGAGGAGATAAGTCTAAGA
